# Novel, Contrast Echocardiography-Based Trabeculation Quantification Method in the Diagnosis of Left Ventricular Excessive Trabeculation

**DOI:** 10.3390/jimaging12040169

**Published:** 2026-04-14

**Authors:** Kristóf Attila Farkas-Sütő, Balázs Mester, Flóra Klára Gyulánczi, Krisztina Filipkó, Hajnalka Vágó, Béla Merkely, Andrea Szűcs

**Affiliations:** Heart and Vascular Centre, Semmelweis University, 1122 Budapest, Hungary

**Keywords:** left ventricular excessive trabeculation, trabeculae, noncompaction, echocardiography, contrast-enhanced echocardiography, diagnostics

## Abstract

Cardiac MRI (CMR) is the gold standard for diagnosing left ventricular excessive trabeculation (LVET), whereas echocardiography (Echo) often does not yield a definitive diagnosis. The use of ultrasound contrast material offers the potential for more accurate imaging of the trabecular system; however, we do not yet have diagnostic criteria developed specifically for contrast Echo (CE-Echo). We aimed to determine the role of CE-Echo in the diagnosis of LVET and to propose a novel method for quantifying trabeculation. We included 55 LVET subjects and 54 age- and sex-matched healthy Control subjects. All subjects underwent non-contrast Echo, CE-Echo, and CMR examinations. In addition to volumetric parameters and ejection fraction (EF), we measured the area of the trabeculated layer and its ratio to the LV area (Trab/LV_area) on apical CE-Echo views. Based on the CMR-derived diagnosis, the Trab/LV_area ratio identified individuals with LVET with high specificity (98%) and sensitivity (95%) when the average of the apical views reached 17% (AUC = 0.98), or when it exceeded 20% in at least one view (AUC = 0.96). The use of CE-Echo may assist in the quantitative diagnosis of LVET in addition to its morphological assessment, and the Trab_area/LVarea may be a good additional criterion in the diagnosis of LVET.

## 1. Introduction

Primary left ventricular excessive trabeculation (LVET) is a distinct phenotype associated with a broad spectrum of left ventricular function and clinical manifestations, ranging from asymptomatic, preserved function to heart failure complicated by arrhythmia or stroke [1,2].

Although the 2023 European Society of Cardiology (ESC) guideline on cardiomyopathies introduced a paradigm shift in the nomenclature of LVET, the previously established noncompacted-to-compacted myocardium (NC/C) ratio-based diagnostic criteria for noncompaction remained unchanged [3]. Nowadays, cardiac magnetic resonance imaging (CMR) serves as the gold standard for diagnosis by using the Petersen and Jaquier criteria; however, the Jenni and Chin echocardiographic (Echo) diagnostic criteria can also be used in cases of good image quality [4,5,6,7]. In many cases, with a suboptimal acoustic window, it can be challenging to obtain a good-quality, non-oblique short axis (SAX) image or an apical view without near-field artefacts on which the apical noncompacted (NC) and compact (C) layers can be clearly distinguished.

Contrast-enhanced echocardiography (CE-Echo) improves visualization and overcomes apical near-field artefacts, thereby enabling a more precise measurement of NC myocardium. Previous case reports and studies show that contrast agents enhance the diagnostic accuracy of Echo in LVET [8,9,10,11,12,13,14]. In these studies, the CE-Echo diagnosis relied on either qualitative assessment alone or quantitative measures using the Jenni and Chin cut-off values; however, those values were established using non-contrast (N) images [13]. Furthermore, Zhang et al. demonstrated that the NC and C layer thickness measurements, the basis of these diagnostic criteria, differ significantly between N-Echo and CE-Echo, raising questions about the interchangeable use of these criteria across the two Echo modalities [15,16].

Evidence from previous studies prompted the European and American imaging societies to recommend the use of contrast media for evaluating noncompaction; however, these recommendations are limited to qualitative assessment, as no CE-Echo–specific quantitative diagnostic criteria have been defined to date [17,18].

Thus, we hypothesized that, with CE-Echo, the extent of trabeculation can be quantified by tracing its area (Trab_area) on apical views, thereby helping to differentiate between LVET and normal controls.

In our study, we aimed to compare the volumetric, functional, and Trab_area parameters between an LVET and a healthy control group, investigate the association between CMR-derived trabecular mass and CE-Echo-derived Trab_area, and determine whether this novel CE-Echo trabecula quantification method could serve as an accurate diagnostic tool.

## 2. Materials and Methods

In our prospective study, all participants underwent CMR and transthoracic echocardiography on the same day.

### 2.1. Study Population

Our team maintains a detailed database of individuals with LVET morphology diagnosed by CMR. In our prospective study, we enrolled 55 (23 female) LVET individuals from this database fulfilling both the Petersen (noncompacted to compacted myocardial layer thickness ≥ 2.3) and the Jacquier (trabecular mass to total myocardial mass ≥ 20%) criteria for noncompaction (Petersen, Jacquier). Exclusion criteria were congenital, ischemic, or significant valvular heart diseases; untreated hypertension; other cardiomyopathies; sports activity exceeding 6 h per week; relevant extracardiac comorbidities and conditions that can cause secondary hypertrabeculation, or patients whose CMR or Echo images were not usable due to technical reasons. Since the imaging criteria alone are prone to overdiagnosing LVET, each of our subjects also underwent a rigorous risk stratification based on the algorithm proposed by Vergani et al. and Negri et al., following the red flag system introduced by Grebur et al., and excluded low-risk individuals without any risk factors [2,19].

Alarming red flags included reduced LVEF and LV dilatation documented with any imaging modality, late gadolinium enhancement (LGE) on CMR images, pathogenic mutation or positive family history, documented ventricular arrhythmias, depolarization or repolarization abnormalities on ECG, thromboembolic events, and unexplained syncope (Table S1).

For the control (C) group, we selected a population of similar age and sex as the LVET group (54 individuals, 22 female). We carefully reviewed the anamnestic data of the population and included only individuals without significant comorbidities (e.g., untreated hypertension, ischemic heart disease, cardiomyopathy, congenital anomalies, extracardiac disorders) and without significant abnormalities on cardiac MRI, and with <6 h/week of sports activity.

The baseline characteristics of the two populations are described in Table 1. Although the mean of the volumetric and functional parameters of the LVET group falls within the normal limits, it is worth mentioning that 9 (16.4%) subjects had reduced LVEF, and 26 (47.3%) had LV dilation (Table S1).

All procedures performed in this study were in accordance with the 1964 Declaration of Helsinki and its subsequent amendments, or with comparable ethical standards. Ethical approval was obtained from the Medical Research Council of Hungary and issued by the National Institute of Pharmacy and Nutrition. All participants provided informed written consent. The patient data, including the ECG recordings, CMR examinations, genetic results, and medical records, were collected from April 2024 to May 2025. All data that could identify individuals in the study were only accessed by K.A.F.-S. and A.S.

### 2.2. Echocardiographic Imaging

Transthoracic 2D Echo was performed on all participants in a supine position using GE Vivid E95 ultrasound machines (GE HealthCare Technologies Inc., Chicago, IL, USA) and a 4Vc phased-array matrix transducer. ECG-gated cine loops were recorded in breath-pause of the standard apical four-, three-, and two-chamber (A4Ch, A3Ch, and A2Ch, respectively) views and SAX views at the level of the mitral valve, papillary muscles, and the apex. All cine loops were first recorded before the contrast injection and then repeated after the intravenous contrast agent was administered, and an equilibrium state/homogenous LV endocardium delineation had been reached. We used a 0.6 mL bolus injection of SonoVue with a 10 mL saline flush. The bolus injection was repeated when apical swirling started to appear; on average, three bolus injections were needed to achieve optimal image quality and record all the necessary apical and SAX views. A contrast-specific imaging preset was used, with the mechanical index (MI) set to 0.21, compression and gain set to optimal trabecular visualization. Image acquisition was timed after the basal attenuation artefact had resolved, but before the prominent apical swirling precluded reliable visualization of the trabeculation. Additionally, a higher, 0.27 MI setting was used when the trabeculation was barely visible at the standard setting.

Offline image analysis was performed using Philips Ultrasound Workspace (version 6.0.0.0, Royal Philips, Amsterdam, The Netherlands). Volumetric measurements were derived from LV endocardial contours at the border between NC and C myocardium on A4Ch and A2Ch views. The non-contrast images were analyzed using the LV-Autostrain module, which features semi-automatic endocardial contour tracing throughout the entire myocardial cycle, allowing for manual adjustment in end-diastole and end-systole. On the CE images, the endocardium was manually traced on the end-diastolic and end-systolic frames only, as the software is unable to process CE-Echo cine loops. Using these contours, the software calculated the end-diastolic, end-systolic, and stroke volumes (EDV, ESV, SV) and the EF via Simpson’s biplane method.

**Figure 1:**
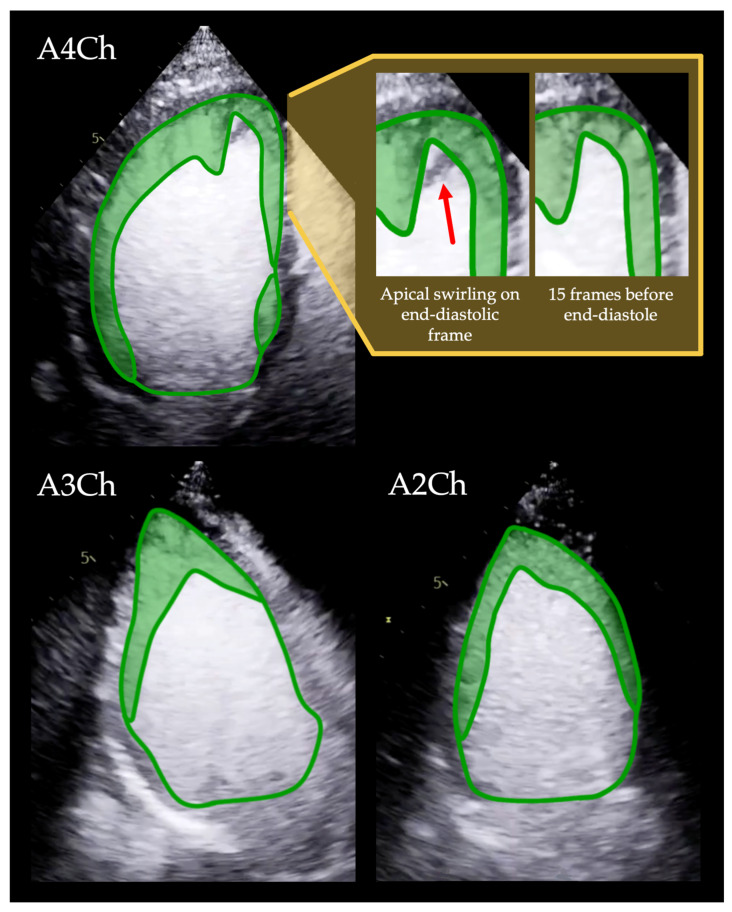
Trab_area tracing method using CE-Echo. The green lines show the Trab_area and LV_area tracings in the apical 4-3-2 chamber views, with the trabeculated area highlighted in light green. Before the yellow background, the apex is shown in the apical four-chamber view. The red arrow indicates an apical swirling artefact visible on the end-diastolic frame, which closely resembles trabeculation on the still image. However, it is not present 15 frames before the end-diastolic frame, confirming that it is an artefact rather than true trabeculation. Video S1. demonstrated the same cine loop, where the artefact’s transient appearance is clearly identifiable. This underscores the importance of assessing trabecular patterns in moving cine loops rather than relying solely on static frames. The video material for the same tracings, the apical 3 and 2-chamber views, can be seen in Videos S2 and S3, respectively. Note that in the 4-chamber view, two unconnected trabeculated regions are traced separately and combined to obtain the overall Trab_area in that view. Abbreviations: A4-3-2Ch: apical 4-3-2 chamber views; CE-Echo: contrast-enhanced echocardiography; LV_area: the area of the left ventricle; Trab_area: the area of the trabeculated layer.

To quantify trabeculation, we developed a novel parameter, the trabeculated area (Trab_area), measured on end-diastolic apical 4-, 3-, and 2-chamber long-axis images. Trab_area was obtained by tracing the endocardial contour along the boundary between the C and NC myocardial layers and following the intracavitary outline of the trabeculations, as illustrated in Figure 1.

**Figure 2:**
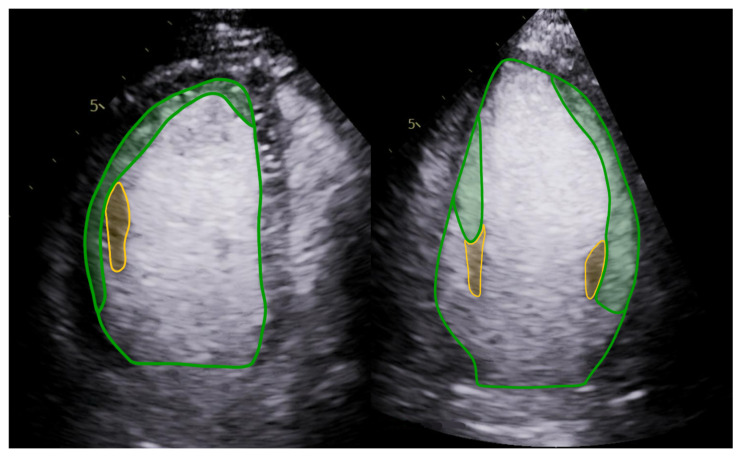
Trab_area tracing with papillary muscles. The Trab_area tracing includes the trabeculated basal part of the papillary muscles (green highlight) up to the point where it appears compact (yellow highlight). Abbreviations: Trab_area: the area of the trabeculated layer.

Because the bases of the papillary muscles are typically trabeculated, they were included in the Trab_area until the point where the papillary muscle appeared compact and clearly separable from the NC layer (Figure 2).

For reference, the left ventricular area (LV_area) was measured in the same apical views by tracing the contour along the border between the C and NC myocardium and connecting the hinge points of the mitral valve leaflets at the annulus, similarly to the volumetric tracings. In the A3Ch view, the aortic annulus and the mitral leaflet insertion points defined the basal contour boundary (Figure 1).

During analysis, tracings were performed on the end-diastolic frame of the cine images, and then their accuracy was subsequently verified by reviewing the corresponding cine loops, which facilitated assessment of the trabecular layer and distinction from apical swirling artefacts [20]. Based on the cine review, the tracings were adjusted as necessary.

When 0.21 MI imaging was insufficient to define the trabecular pattern, 0.27 MI recordings were also used. Both 0.21 and 0.27 MI images were examined in parallel to accurately assess the trabecular architecture, with the final Trab_area traced on the lower MI image.

From these tracings, the Trab/LV_area ratio (expressed as a percentage) was calculated for each A4-, A3-, and A2Ch view separately. From the three apical views, both the mean (avg) and maximum (max) values of Trab_area and the Trab/LV_area ratio were computed for each subject.

In the Discussion (Section 4), we further described our experience with the novel quantification method and summarized our recommendations for tracing trabeculation.

Volumetric and Trab_area parameters were indexed (i) to body surface area (BSA). The interobserver variability of the measured Echo parameters is shown in Table S2, demonstrating excellent reproducibility.

Finally, we have measured the NC/C ratio on non-contrast apical SAX images in end-systole to compare the sensitivity and specificity of our CE-Echo diagnostic criteria with the gold standard N-Echo Jenni criteria (end-systolic NC/C layer thickness ≥ 2) [6].

### 2.3. CMR Image Acquisition and Analysis

The CMR images were acquired by using a Siemens Magnetom Aera (Siemens Healthineers, Erlangen, Germany) 1.5 T MR imaging machine on the same day, within 2 h of the Echo examinations. Our protocol included conventional four-, three-, and two-chamber long-axis cine loops, and SAX cine loops covering the whole LV. We recorded breath-hold images by using retrospectively gated balanced steady-state free precession sequences. The slice thickness was 8 mm without an interslice gap, and the field of view was 350 mm on average, adapted to the body size.

The cine loops were analyzed by using the Medis Suite software (Medis Suite QMass, version 4.0, Medis Medical Imaging Systems, Leiden, The Netherlands).

During post-processing, the endocardial and epicardial contours were delineated on the SAX images in both the end-diastolic and end-systolic frames. The software uses a semi-automatic tracing technique with manual corrections to ensure accurate alignment of the endocardial and epicardial borders. Based on the established contours, the software calculates the LV EDV, ESV, SV, and EF. In order to assess the trabecular and papillary muscle mass (Trab_mass), a threshold-based algorithm was employed. The MassK module analyzes the signal intensity pattern using the 50% threshold setup, differentiating between myocardial and blood tissue. Therefore, the myocardial tissue within the endocardial border represents Trab_mass, measured on the end-diastolic frame. All volumetric and myocardial mass parameters were indexed (i) to the BSA. For reference values, we used the normal ranges established by the Society for Cardiovascular Magnetic Resonance in 2025 [21].

### 2.4. Statistical Analysis

All continuous parameters are reported as the mean with the lower and upper limits of the confidence interval, and all discrete parameters are reported as counts or percentages. The Kolmogorov–Smirnov test was used to assess the normal distribution of continuous parameters, and Levene’s test was used to assess homogeneity.

First, we compared the two Echo modalities within the LVET and Control groups separately using a paired-samples *t*-test and Pearson’s correlation coefficient.

To compare means between the LVET and Control populations, we used independent-samples *t*-tests and Mann–Whitney U-tests, depending on the distribution of the dataset. With Pearson’s correlation coefficient, we assessed the relationship between the CMR-derived Trab_mass and the CE-Echo-derived Trab_area_avg and Trab_area_max measurements. To analyze the diagnostic accuracy of the two Trab_area and the two Trab/LV_area parameters relative to the gold standard CMR diagnostic criteria, we examined their receiver operating characteristic (ROC) curves and set optimal cut-off values using the Youden index.

To assess the reproducibility of our measurements, we used the intraclass correlation coefficient (ICC); values above 0.75 were considered excellent agreement.

A *p*-value below 0.05 was considered statistically significant. IBM SPSS Statistics (Version 30.0, Armonk, NY, USA) was used for statistical analyses.

## 3. Results

### 3.1. Comparisons of Imaging Modalities

First, we compared the N-Echo and CE-Echo modalities in the Control and LVET groups separately. In both groups, the EDV(i), SV(i), and EF were significantly higher when measured by CE-Echo than by N-Echo (Table 2). We also found excellent correlations between the corresponding parameters using the two echo modalities (Table 3).

### 3.2. Comparisons of the LVET and Control Populations Using CE-Echo

**Figure 3:**
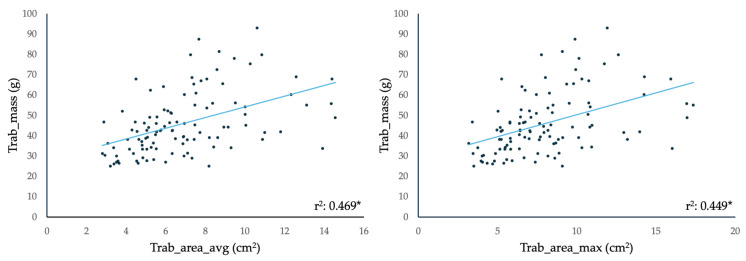
Correlation between the CMR-derived Trab_mass and the CE-Echo-derived Trab_area_avg and Trab_area_max. Abbreviations: *: *p* < 0.05; avg: mean value of the measured parameter, per person; CE-Echo: contrast-enhanced echocardiography; CMR: cardiac magnetic resonance imaging; max: maximum measured value of the parameter, per person; Trab_area: the area of the trabeculated layer; Trab_Mass: mass of the trabeculation and papillary muscles.

We compared the volumetric and functional Echo parameters and the Trab_area measurements on CE-Echo between the LVET and Control populations and found that the LVET group had significantly higher EDV(i), ESV(i), Trab_area(i), and Trab/LV_area, and lower EF (Table 2). When assessing the relationship between the CMR-derived Trab_mass and the CE-Echo-derived Trab_area measurements, we found significant correlations between the two methods of quantifying trabeculation (Figure 3).

**Figure 4:**
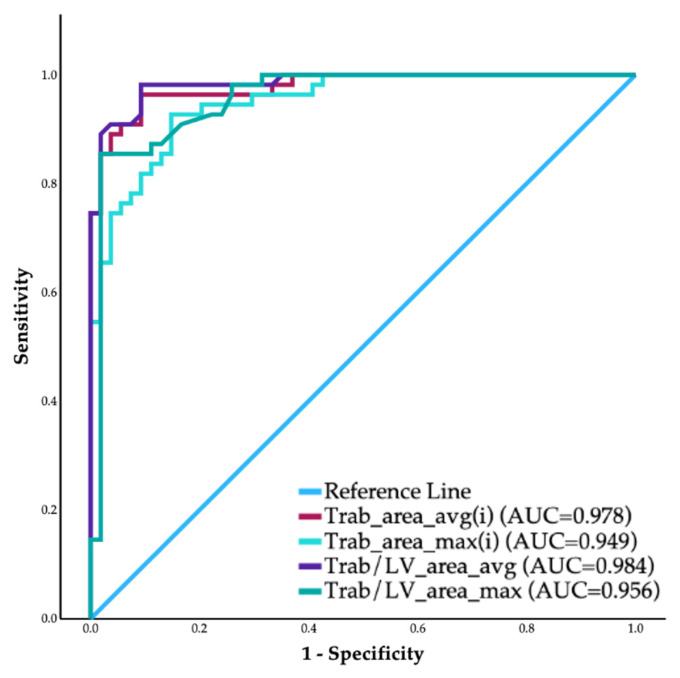
The ROC curves of the CE-Echo-derived trabeculation-quantification parameters. Abbreviations: avg: mean value of the measured parameter, per person; AUC: area under the curve; CE-Echo: contrast-enhanced echocardiography; max: maximum measured value of the parameter, per person; ROC: receiver operator characteristics; Trab_area: the area of the trabeculated layer; Trab/LV_area: the ratio of the Trab_area and the left ventricular area.

Then, we determined whether the Trab_area_avg(i), Trab_area_max(i), or their ratio to the LV_area(i) (Trab/LV_area_avg, Trab/LV_area_max) can be used to correctly classify the LVET subjects. Analyzing the ROC curves of the above-mentioned parameters, the AUC values were 0.977, 0.947, 0.983, and 0.960, respectively (Figure 4).

Based on the Youden indices, we determined the optimal cut-off values for each parameter (Table 4). We analyzed the use of these criteria in combination to further optimize the sensitivity and specificity of the method, keeping clinical usability in mind, and found that if a person has a mean Trab/LV_area ratio of 17% or the ratio reaches 20% in any apical view, the diagnosis of LVET can be established (Table 4). We measured the maximum end-systolic NC: C ratio in our study population and, using the cut-off value of Jenni et al., found that the sensitivity and specificity of the N-Echo were 58.5% and 92.2%, respectively (Table 4).

## 4. Discussion

The diagnosis of LVET is based on imaging modalities, including the gold-standard CMR and the transthoracic Echo. Current diagnostic criteria focus on quantifying the trabecular layer in relation to the compact myocardium, using layer thickness or CMR-derived myocardial mass [4,5,6,7]. Because of the possible life-threatening complications, early diagnosis and risk stratification are key factors in identifying high-risk individuals. The first-line imaging modality is N-Echo, for which several methods have been established to assess left ventricular trabeculation, including the Jenni and Chin (ratio of non-compacted to compacted layer thickness) and the Stöllberger (≥3 prominent trabeculae in the apical view) methods [5,6,22]. All are constrained by image quality, as the border between the NC and C layer is often indistinct, especially on the anterior and anterolateral walls due to lung interference and in the apex due to near-field artefacts. While color Doppler is recommended to confirm intertrabecular flow, its accuracy is also limited by the poor acoustic windows.

For this reason, Echo often does not allow for an accurate diagnosis, and patients require CMR to confirm the LVET. Alternatively, as several previous case studies demonstrated and the most recent CE-Echo guidelines concluded, the use of contrast media enhances the visibility of the border between the NC and C layers, allowing for more precise quantification [8,9,10,11,12,13,14,17,18]. Zhang et al. demonstrated that the previously established N-Echo criteria are not applicable to CE Echo images; however, to date, no published diagnostic LVET criteria have been established for this modality [15,16].

Therefore, this study was designed to establish a CE-Echo-specific diagnostic criterion. We hypothesized that the extent of trabeculation can be quantified by tracing its area on CE-Echo images and that this parameter could serve as a valid diagnostic tool in LVET.

### 4.1. Comparisons of the Two Imaging Modalities

We found higher volumetric measurements with CE-Echo compared to N-Echo, a well-documented phenomenon that has been studied in several populations [17,23,24,25,26,27]. However, it has not been extensively investigated in a hypertrabeculated population, where endocardial tracing is typically more challenging with N-Echo. This probably explains the higher mean intermodality difference in the LVET group compared to the Controls [28].

### 4.2. Comparisons of the LVET and Control Populations

Using CE-Echo, we compared the volumetric, functional, and Trab_area parameters between the LVET and the Control population and found significantly higher volumes and lower EF, which coincides with previously published CMR and N-Echo studies [19,28,29,30,31,32,33,34]. However, we have found no previous studies of CE-Echo comparisons. The Trab_area and Trab/LV_area parameters differed significantly between the two groups, suggesting they could be a promising diagnostic tool to differentiate the LVET population. Moreover, the significant correlation between the CMR-derived Trab_mass and the CE-Echo-derived Trab_area indicates that our novel quantification technique is a representative measure of trabeculation. According to the ROC curves, the average and maximum values of the Trab_area(i) and Trab/LV_area parameters can serve as a viable diagnostic tool; however, to improve diagnostic accuracy, we also tested all sensible combinations of these parameters. Overall, we achieved the best results with the two Trab/LV_area ratios: if a person had either Trab/LV_area_avg ≥ 17.0% or Trab/LV_area_max ≥ 20.0%, the diagnosis of LVET could be established. This combination is also preferable for practical reasons and everyday clinical use: if an individual reaches a 20.0% Trab/LV_area ratio in at least one apical view, the remaining views may not need to be analyzed. This can save time and be useful in cases where optimal image quality cannot be achieved in all apical views (e.g., due to anatomical constraints).

It is worth mentioning that we found overall similarly good specificity and sensitivity when testing the combination of Trab_area_avg(i) and the two Trab/LV_area ratios in the sense that a subject has to reach 3.1 cm^2^/m^2^ Trab_area_avg(i) and one of the cut-off values of the ratios mentioned above to establish the diagnosis. However, since the Trab_area_avg(i) requires the analysis of all three apical views, we considered it less optimal for the reasons stated above.

### 4.3. CE-Echo Imaging and Contouring Insights

Since Trab_area tracing is a novel measurement technique, we aimed to provide more insight into our experience and offer a guide for clinical application.

#### 4.3.1. Image Acquisition

For the evaluation of LV noncompaction, the most recent CE-Echo guideline published by the European Cardiovascular Imaging Association recommends using an intermediate MI setting (0.2–0.5) [17,18,35]. Previous case reports on the topic often lack comprehensive documentation on the MI setting, administration technique, dosage of contrast media, and the model of the ultrasound machine [8,10,11]. Based on the provided images, most studies focused on achieving optimal opacification only in trabeculated regions since the current guidelines prioritize qualitative assessment of noncompacted segments; thus, complete homogeneous opacification of the LV is not necessarily needed once these areas are clearly visualized. However, our quantitative assessment via the Trab_area method can be accurately traced only if there is homogeneous LV enhancement after the basal attenuation has resolved, but before the prominent contrast destruction occurs.

We found two larger cohort studies that investigated the usefulness of CE-Echo in diagnosis, both of which used 0.5 to 1.5 mL bolus injections of SonoVue for LV opacification at an MI between 0.3 and 0.5 [13,15]. Based on the published images, the entire LV was homogeneously opacified, similar to our study; however, they used significantly higher doses of contrast, on average, almost 2 times as much as we did. On our GE Vivid E95 ultrasound machine, we used the available LV Contrast preset with an MI setting of 0.21, which is lower than the previous studies but falls within the lower limit of intermediate MI imaging. In our experience, when we increased the output power, the microbubble destruction and apical swirling artefact were so prominent that adequate visualization of the trabeculation was impossible. This could be attributed to the significantly lower amount of contrast media injected compared to previous studies, or maybe even the vendor of the ultrasound machine can play a part.

In our study, there was one case where the trabeculation of the basal and mid-segment of the anterior wall could not be well visualized in the apical 2-chamber view, and we were unable to determine whether trabeculation was present or it was just an artefact. When we increased the output power, it became apparent that trabeculation was indeed present; however, the apex could not be well visualized due to the significant apical swirling with the higher MI setting. Therefore, we still traced the Trab_area on the lower, 0.21 MI image (Video S4). Aggarwal et al. published a case report describing a similar phenomenon: trabeculation was more visible at higher MI, while apical swirling was more prominent [14].

Based on our experience and the published literature, the contrast agent dose and MI setting should be appropriately matched, with higher MI values requiring larger contrast doses. We recommend tailoring these parameters to the patient’s image quality and the ultrasound machine’s capabilities to achieve optimal trabecular visualization.

To further optimize the image for trabeculation, we found that lowering the 2D gain improved visualization of the trabecular network (Video S5). The GE automatic image enhancement tool can improve image clarity in poor acoustic windows by offering soft and sharp filter settings; however, sometimes the best image quality is achieved without any enhancement.

It is crucial to avoid off-axis and foreshortened images, as they can drastically overestimate the Trab_area and underestimate the LV_area, resulting in an unreasonably large Trab/LV_area ratio and a false-positive diagnosis (Video S6).

Previous CE-Echo studies have suggested using SAX images to assess the extent of apical trabeculation, similar to the N-Echo Jenni criteria. However, in our experience, CE-Echo SAX images in the apical region often do not provide sufficient image quality for quantification; thus, we chose the significantly better and more reliable apical views.

#### 4.3.2. Trabeculation Tracing Methods

The tracing of visualized trabeculae on CE-Echo is a novel quantification technique without any previously established guidelines; therefore, this chapter aims to summarize our experience-based recommendations for the method, in an effort to standardize the measurement.

When tracing the trabeculation, the NC-C border is easily identified using CE-Echo; however, the LV border of the layer can be more challenging to assess, especially when apical swirling develops rapidly around an extensive trabecular layer. Trabeculae create an uneven surface that can be difficult to identify on a still image, making it challenging to differentiate trabeculation from apical swirling (Figure 1) [14]. Therefore, we recommend verifying the tracing on the moving cine loop and making further corrections if the software allows additional editing after the initial tracing. If this feature is unavailable, side-by-side comparison of static and cine images from the same view, tracing on the static frame while using the cine loop as additional reference, can also aid accuracy.

In the following paragraphs, we would like to introduce the specific circumstances we encountered while tracing the noncompacted layer. In general, most people have some degree of traceable trabeculation in the apical region, as well as along the sides of the anterior and lateral walls, forming a continuous meshwork that can be traced in one contour. However, the overall pattern of trabeculation varies from person to person, particularly in individuals with LVET. Occasionally, we observed trabeculation in two separate, unconnected segments of the left ventricle, resulting in two separate tracings that added up to the overall Trab_area. Although this is also present in healthy individuals and is not a phenomenon specific to LVET (Figure 1, Video S1).

When the trabeculae blended together to form a network, we covered the surface of the mashwork with the tracing; however, sizable intertrabecular gaps were excluded from the tracings (Figure 1, Video S1). On rare occasions, a sizable trabecula can connect two opposite walls of the left ventricle, resulting in extraordinary tracing patterns as demonstrated in Video S7. Thin false tendons, however, are usually overshadowed by the high echogenicity of the contrast media, making them practically invisible on CE-Echo.

The origin and often the basal portion of the papillary muscles are usually trabeculated; therefore, to ensure the best possible reproducibility, we traced them to the Trab_area up to the point where it appeared to be a compact muscle. It should be noted that, since CE-Echo has a lower spatial resolution in the far field, visualization of trabeculation in the basal region of the LV can sometimes be challenging, particularly near the papillary muscles. In these challenging cases, the previously mentioned higher-MI imaging can enhance visualization beyond conventional image optimization techniques. Finally, even if the fine structure of the trabecular meshwork cannot be fully appreciated, the overall extent of trabeculation can still be estimated based on echogenicity.

Calcified hyperechogenic trabeculae can be challenging to visualize with CE-Echo, as they exhibit a very similar echogenicity to the contrast media (Video S8). These cases highlight the importance of integrating N-Echo and CE-Echo in assessing trabecular patterns, as hyperechogenic trabeculae should be visible without contrast media. Zhang et al. also reported that, although CE-Echo improved LVET diagnosis and was superior to N-Echo, the best results were achieved when combining the two Echo modalities [15]. We also had several cases where we compared the N-Echo and CE-Echo cine loops side by side to gain a comprehensive understanding of the trabecular pattern.

Overall, our study highlighted the utility of CE_Echo in the quantitative diagnosis of LVET, as the Trab/LV area ratio has proven to be an accurate diagnostic parameter.

### 4.4. Limitation

Our study had a lower representation of individuals with severely dilated LV, which would significantly elevate the LV_area, thereby affecting the Trab/LV_area ratio and potentially reducing sensitivity in that population. However, several dilated LVs usually indicate a hypertrabeculated DCM overlapping phenotype, suggesting that the diagnosis of LVET carries less prognostic value, and CMR imaging is part of the risk stratification and can diagnose LVET.

The relatively low number of cases also limits our study; however, since high-risk LVET is a rare condition, the size of our population matches that of similar LVET studies previously published. In the future, we plan to continue this study to further optimize the CE-Echo diagnostic methods for LVET.

## 5. Conclusions

In our study, we aimed to establish a CE-Echo-specific LVET diagnostic criterion based on tracing the Trab_area on apical views. We also compared the N-Echo and CE-Echo modalities in an LVET and a Control group separately and assessed the difference in volumetric and functional parameters between the two populations.

While the two Echo modalities show a strong correlation, we found higher volumes and LVEF values with CE-Echo than with N-Echo.

Using CE-Echo, LVET subjects demonstrated higher LV volumes and Trab_area, and lower ejection fraction than Controls, and CE-derived trabecular areas correlated with CMR-derived trabecular mass, supporting CE-Echo as a robust tool for quantifying trabeculation. ROC analysis demonstrated that trabecular area and the Trab/LV_area ratio had high sensitivity for detecting LVET, with a practical diagnostic threshold defined as a mean Trab/LV_area ≥17% or Trab/LV_area ≥ 20% in any apical view.

Overall, our findings indicate that CE-Echo–based Trab/LV_area metrics can reliably reproduce the CMR-based classification of LVET. It may offer a more accessible, ultrasound-based reference for detecting pathological LV trabeculation in routine clinical practice, while highlighting the need for further validation in larger and more diverse cohorts.

## Figures and Tables

**Table 1:** Baseline characteristics of the studied populations using CMR.

	LVET	Control	*p*
**n (% females)**	55 (42%)	54 (41%)	n.s.
**Age (years)**	39.5 [35.4–43.5]	41.5 [37.2–45.8]	0.488
**BSA (m^2^)**	1.95 [1.88–2.02]	1.97 [1.90–2.05]	0.687
**CMR_EDV(i) (m** **L** **/m^2^)**	97.1 [92.2–101.9]	82.8 [79.7–85.9]	<0.001
**CMR_ESV(i) (m** **L** **/m^2^)**	44.1 [41.1–47.1]	33.6 [31.9–35.3]	<0.001
**CMR_SV(i) (m** **L** **/m^2^)**	53.0 [50.5–55.5]	49.2 [47.3–51.1]	0.016
**CMR_EF (%)**	54.9 [53.7–56.2]	59.5 [58.3–60.6]	<0.001
**CMR_Trab_mass (g/m^2^)**	26.3 [24.4–28.2]	20.2 [19.2–21.2]	<0.001

Values presented as Mean [lower and upper limits of confidence interval]. Abbreviations: BSA: body surface area; CMR: cardiac magnetic resonance imaging; EDV: end diastolic volume; EF: ejection fraction; ESV: end systolic volume; i: indexed to body surface area; LVET: left ventricular excessive trabeculation; n: number of subjects; n.s.: non-significant; SV: stroke volume; Trab_mass: trabeculated and papillary muscle mass.

**Table 2:** N-Echo and CE-Echo parameters of the LVET and Control populations.

	N-Echo		CE-Echo		N-Echo vs. CE-Echo		LVET vs. Control	
					LVET	Control	N-Echo	CE-Echo
	LVET	Control	LVET	Control	*p*	*p*	*p*	*p*
**n**	55	54	55	54	-	-	-	-
**EDV(i)** **(mL/m^2^)**	69.6 [65.8–73.4]	60.8 [57.9–63.8]	78.2 [73.8–82.7]	66.7 [63.5–70.0]	<0.001	<0.001	<0.001	<0.001
**ESV(i)** **(mL/m^2^)**	29.9 [27.8–32.0]	22.9 [21.6–24.3]	32.2 [29.6–34.8]	23.4 [22.0–24.8]	<0.001	0.107	<0.001	<0.001
**SV(i)** **(m** **L** **/m^2^)**	39.4 [37.2–41.6]	37.9 [36.0–39.8]	46.0 [43.6–48.5]	43.3 [41.1–45.4]	<0.001	<0.001	0.074	0.083
**EF** **(%)**	57.3 [56.1–58.5]	62.3 [61.2–63.5]	59.3 [57.7–60.9]	64.9 [63.8–66.0]	<0.001	<0.001	<0.001	<0.001
**Trab_area_avg(i)** **(cm^2^/m^2^)**	-	-	4.6 [4.2–4.9]	2.5 [2.3–2.6]	-	-	-	<0.001
**Trab_area_max(i)** **(cm^2^/m^2^)**	-	-	5.2 [4.8–5.6]	3.0 [2.8–3.1]	-	-	-	<0.001
**Trab/LV_area_avg** **(%)**	-	-	22.6 [21.3–24.0]	13.4 [12.7–12.1]	-	-	-	<0.001
**Trab/LV_area_max** **(%)**	-	-	25.4 [23.8–26.9]	15.7 [14.7–16.7]	-	-	-	<0.001

Values presented as Mean [lower and upper limits of confidence interval]. Abbreviations: avg: mean value of the measured parameter, per person; CE-Echo: contrast-enhanced echocardiography; EDV: end diastolic volume; EF: ejection fraction; ESV: end systolic volume; i: indexed to body surface area; LVET: left ventricular excessive trabeculation; max: maximum measured value of the parameter, per person; n: number of subjects; N-Echo: non-contrast echocardiography; SV: stroke volume; Trab_area: the area of the trabeculated layer; Trab/LV_area: the ratio of the trabeculated area and the LV area.

**Table 3:** Correlation between the three imaging modalities in the studied population.

**LVET**								
**Correlation Coefficient**	**EDV_N-Echo**	**ESV_N-Echo**	**SV_N-Echo**	**EF_N-Echo**	**EDV_CMR**	**ESV_CMR**	**SV_CMR**	**EF_CMR**
**EDV_CE-Echo**	0.932 **	0.840 **	0.868 **	−0.160	0.891 **	0.848 **	0.790 **	−0.254
**ESV_CE-Echo**	0.859 **	0.923 **	0.663 **	−0.495 **	0.831 **	0.888 **	0.640 **	−0.474 **
**SV_CE-Echo**	0.810 **	0.597 **	0.876 **	0.171	0.765 **	0.642 **	0.764 **	−0.007
**EF_CE-Echo**	−0.311 *	−0.558 **	−0.029	0.730 **	−0.341 *	−0.503 **	−0.124	0.569 **
**Control**								
**Correlation Coefficient**	**EDV_N-Echo**	**ESV_N-Echo**	**SV_N-Echo**	**EF_N-Echo**	**EDV_CMR**	**ESV_CMR**	**SV_CMR**	**EF_CMR**
**EDV_CE-Echo**	0.904 **	0.815 **	0.876 **	−0.172	0.879 **	0.804 **	0.823 **	−0.249
**ESV_CE-Echo**	0.853 **	0.882 **	0.740 **	−0.436 **	0.831 **	0.858 **	0.690 **	−0.468 **
**SV_CE-Echo**	0.848 **	0.689 **	0.879 **	0.024	0.825 **	0.690 **	0.830 **	−0.080
**EF_CE-Echo**	−0.314 *	−0.540 **	−0.107	0.709 **	−0.307 *	−0.492 **	−0.100	0.613 **

Volumetric parameters were measured in ml, EF is in percentage. Abbreviations: **: Correlation is significant at the 0.01 level; *: Correlation is significant at the 0.05 level; CE-Echo: contrast-enhanced echocardiography; CMR: cardiac magnetic resonance imaging; EDV: end diastolic volume; EF: ejection fraction; ESV: end systolic volume; i: indexed to body surface area; LVET: left ventricular excessive trabeculation; N-Echo: non-contrast echocardiography; SV: stroke volume; background color and bold, enhances readability.

**Table 4:** Correlation between the imaging modalities in the studied populations.

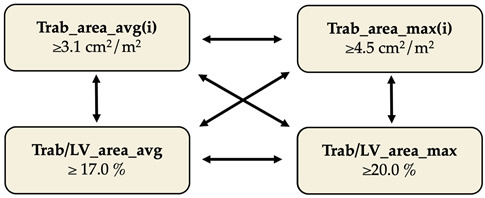				
	**Sensitivity**	**Specificity**	**PPV**	**NPV**
**Single criterion**				
Trab_area_avg(i) ≥ 3.1 cm^2^/m^2^	0.96	0.91	0.91	0.96
Trab_area_max(i) ≥ 4.5 cm^2^/m^2^	0.92	0.83	0.85	0.92
Trab/LV_area_avg ≥ 17.0%	0.89	0.98	0.98	0.90
Trab/LV_area_max ≥ 20.0%	0.85	0.98	0.98	0.87
**Sensible combinations that can possibly improve accuracy**				
Either of the four criteria	0.98	0.81	0.84	0.98
All four criteria	0.75	1.00	1.00	0.79
**Trab/LV_area_avg OR Trab/LV_area_max**	**0.95**	**0.98**	**0.98**	**0.95**
Trab/LV_area_avg AND Trab/LV_area_max	0.80	0.98	0.98	0.83
Trab_area_avg(i) OR Trab/LV_area_avg	0.98	0.89	0.90	0.98
Trab_area_avg(i) OR Trab/LV_area_max	0.98	0.89	0.90	0.98
Trab_area_avg(i) AND Trab/LV_area_avg	0.87	1.00	1.00	0.89
Trab_area_avg(i) AND Trab/LV_area_max	0.84	1.00	1.00	0.86
Trab_area_avg(i) OR either Trab/LV_area ratio	0.98	0.89	0.90	0.98
Trab_area_avg(i) AND either Trab/LV_area ratio	0.93	1.00	1.00	0.93
Trab_area_avg(i) AND both Trab/LV_area ratio	0.78	1.00	1.00	0.82
**N-Echo Jenni Criteria**				
NC/C ratio ≥ 2.0	0.58	0.92	0.89	0.68

Abbreviations: avg: mean value of the measured parameter, per person; i: indexed to body surface area; max: maximum measured value of the parameter, per person; N-Echo: non-contrast echocardiography; Trab_area: the area of the trabeculated layer; Trab/LV_area: the ratio of the trabeculated area and the LV area; background color and bold, enhances readability.

## Data Availability

The original data presented in this study are included in the article and Supplementary Materials. Further inquiries can be directed to the corresponding authors.

## Supplementary Materials

The following supporting information can be downloaded at: https://www.mdpi.com/article/10.3390/jimaging12040169/s1. Table S1: Clinical characteristics of the LVET population; Table S2: Interobserver variabilities of the studied Echo parameters; Video S1: Trab_area tracing in apical 4 chamber view using CE-Echo; Video S2. Trab_area tracing in apical 3-chamber view using CE-Echo; Video S3. Trab_area tracing in apical 2-chamber view using CE-Echo; Video S4: The difference between 0.21 and 0.27 MI; Video S5: The appearance of trabeculation with different gain settings; Video S6: The importance of avoiding foreshortening; Video S7: The noncontrast and contrast echocardiography recordings of a subject with extreme trabeculation and unusual trabecular pattern; Video S8: Hyperechoic trabeculation.

## Abbreviations

The following abbreviations are used in this manuscript:

A2Ch	Apical two-chamber view
A3Ch	Apical three-chamber view
A4Ch	Apical four-chamber view
AUC	Area under the curve
BSA	Body surface area
C	Compact myocardial layer
CE-Echo	Contrast-enhanced echocardiography
CMR	Cardiac magnetic resonance imaging
DCM	Dilated cardiomyopathy
Echo	Echocardiography
ECG	Electrocardiogram
EDV	End-diastolic volume
EF	Ejection fraction
ESC	European Society of Cardiology
ESV	End-systolic volume
i	Indexed to body surface area (used as a suffix)
ICC	Intraclass correlation coefficient
LGE	Late gadolinium enhancement
LV	Left ventricle/ventricular
LV_area	Left ventricular area in apical view
LVET	Left ventricular excessive trabeculation
MI	Mechanical index
N-Echo	Non-contrast echocardiography
NC	Non-compacted myocardial layer
NC/C	Non-compacted-to-compacted myocardium ratio
NPV	Negative predictive value
PPV	Positive predictive value
ROC	Receiver operating characteristic
SAX	Short-axis view
SV	Stroke volume
Trab_area	Area of the trabeculated layer
Trab/LV_area	Ratio of trabeculated area to LV area
Trab_mass	Trabecular and papillary muscle mass
