# Supplementary material for: Novel, Contrast Echocardiography-Based Trabeculation Quantification Method in the Diagnosis of Left Ventricular Excessive Trabeculation
Source: J Imaging. 2026 Apr 14;12(4):169. doi: 10.3390/jimaging12040169 (PMC13117673; doi:10.3390/jimaging12040169)
Supplement: Supplementary file 1 [file jimaging-12-00169-s001.zip › jimaging-4209783-supplementary/Supplementary material/Table S1.pdf]

Table S1. Clinical characteristics of the LVET population

Red-flag	Number of subjects (%)
**LV dilation ever recorded**	29 (52,7%)
**LV dilation in the current study**	26 (47,3%)
**Reduced LV EF ever recorded**	14 (25,5%)
**Reduced LV EF in the current study**	9 (16,4%)
**LGE**	8 (14,5%)
**Pathogenic mutation/Genetic mutation**	6 (10,9%)
**Positive family history**	20 (36,4%)
**Documented arrhythmia**	14 (25,5%)
**Supraventricular**	8 (14,5%)
**Ventricular**	10 (18,2%)
**Depolarization abnormality**	20 (36,4%)
**Repolarization abnormality**	3 (5,5%)

Abbreviations: LGE: Late gadolinium enhancement; LV: left ventricle; LVET: left ventricualar excessive trabeculation
