# Supplementary material for: Novel, Contrast Echocardiography-Based Trabeculation Quantification Method in the Diagnosis of Left Ventricular Excessive Trabeculation
Source: J Imaging. 2026 Apr 14;12(4):169. doi: 10.3390/jimaging12040169 (PMC13117673; doi:10.3390/jimaging12040169)
Supplement: Supplementary file 1 [file jimaging-12-00169-s001.zip › jimaging-4209783-supplementary/Supplementary material/Table S2.pdf]

Table S2. Interobserver variabilities of the studied parameters

	ICC (LLC-ULC)
**N-Echo**	
**EDV(i) (ml/m²)**	0,982 (0,927-0,995)
**ESV(i) (ml/m²)**	0,968 (0,871-0,992)
**SV(i) (ml/m²)**	0,957 (0,829-0,989)
**EF (%)**	0,789 (0,152-0,948)
**CE-Echo**	
**EDV(i) (ml/m²)**	0,989 (0,989-0,999)
**ESV(i) (ml/m²)**	0,985 (0,932-0,997)
**SV(i) (ml/m²)**	0,945 (0,757-0,988)
**EF (%)**	0,936 (0,718-0,986)
**Trab_area(i)**	0,989 (0,951-0,998)
**Trab/LV_area**	0,987 (0,942-0,997)

ICC ≥ 0.75 Excellent agreement; 0.75 > ICC > 0.4 Fair to good agreement; ICC ≤ 0.4 Poor agreement
Abbreviations: CE-Echo: contrast-enchanced echocardiography; EDV: end diastolic volume; EF: ejection fraction; ESV: end systolic volume; i: indexed to body surface area; ICC: intraclass correlation coefficient; N-Echo: non-contrast echocardiography; SV: stroke volume; Trab_area: the area of the trabeculated layer; Trab/LV_area: the ratio of the trabeculated area and the LV area
