# Supplementary material for: Novel, Contrast Echocardiography-Based Trabeculation Quantification Method in the Diagnosis of Left Ventricular Excessive Trabeculation
Source: J Imaging. 2026 Apr 14;12(4):169. doi: 10.3390/jimaging12040169 (PMC13117673; doi:10.3390/jimaging12040169)
Supplement: Supplementary file 1 [file jimaging-12-00169-s001.zip › jimaging-4209783-supplementary/Supplementary material/Vdeos/Vdeo legends.pdf]

Video S1. Trab_area tracing in apical 4-chamber view using CE-Echo. The green lines show the Trab_area and LV_area tracings in the apical 4-chamber view, with the trabeculated area highlighted in light green. Note that in the 4-chamber view, two unconnected trabeculated regions are traced separately and combined to obtain the overall Trab_area in that view.
Abbreviations: CE-Echo: contrast-enhanced echocardiography; LV_area: the area of the left ventricle; Trab_area: the area of the trabeculated layer

Video S2. Trab_area tracing in apical 3-chamber view using CE-Echo. The green lines show the Trab_area and LV_area tracings in the apical 3-chamber view, with the trabeculated area highlighted in light green.
Abbreviations: CE-Echo: contrast-enhanced echocardiography; LV_area: the area of the left ventricle; Trab_area: the area of the trabeculated layer

Video S3. Trab_area tracing in apical 2-chamber view using CE-Echo. The green lines show the Trab_area and LV_area tracings in the apical 2-chamber view, with the trabeculated area highlighted in light green.
Abbreviations: CE-Echo: contrast-enhanced echocardiography; LV_area: the area of the left ventricle; Trab_area: the area of the trabeculated layer

Video S4. The difference between 0.21 and 0.27 MI. The trabeculation along the anterior wall is barely visible on the lower MI image (left) but is clearly visible on the higher MI image (right). Note the prominent apical swirling on the higher MI loop.
Abbreviations: MI: mechanical index

Video S5. The appearance of trabeculation with different gain settings. The video shows the apical 4-chamber view of a subject with excessive left ventricular trabeculation at higher (left) and lower (right) gain settings, demonstrating that the trabecular layer is more visible at lower gain.

Video S6. The importance of avoiding foreshortening. On the left, a foreshortened apical 3-chambered view shows prominent trabeculation at the apical anteroseptal segment; however, this is not visible when imaging from the true apex.

Video S7. The noncontrast (left) and contrast (right) echocardiography recordings of a subject with extreme trabeculation and an unusual trabecular pattern

Video S8. Hyperechoic trabeculation. The video shows the apical 4-chamber view of a subject with excessive left ventricular trabeculation, with noncontrast and contrast images side-by-side. The bright echogenic trabeculae in the apical region of the inferior septum are clearly visible on noncontrast imaging (right); however, they are masked when contrast media is injected (right). At a later stage of the examination, when the contrast media wears out, the echogenic trabeculae are visible again. This highlights the importance of integrating noncontrast and contrast images to obtain a comprehensive understanding of the trabecular pattern.
